# Differences in training characteristics of recreational endurance runners by race distance – results from the NURMI Study (Step 2)

**DOI:** 10.3389/fpsyg.2023.1269374

**Published:** 2024-01-09

**Authors:** Beat Knechtle, Derrick Tanous, Mabliny Thuany, Mohamad Motevalli, Gerold Wirnitzer, Claus Leitzmann, Katja Weiss, Thomas Rosemann, Katharina Wirnitzer

**Affiliations:** ^1^Institute of Primary Care, University of Zurich, Zurich, Switzerland; ^2^Medbase St. Gallen Am Vadianplatz, St. Gallen, Switzerland; ^3^Department of Sport Science, University of Innsbruck, Innsbruck, Austria; ^4^Department of Research and Development in Teacher Education, University College of Teacher Education, Innsbruck, Tyrol, Austria; ^5^Faculty of Sports, University of Porto, Porto, Portugal; ^6^AdventureV & Change2V, Stans, Austria; ^7^Institute of Nutrition, University of Gießen, Gießen, Germany; ^8^Research Center Medical Humanities, Leopold-Franzens University of Innsbruck, Innsbruck, Austria

**Keywords:** 10 kilometers, half-marathon, marathon, ultra-marathon, running, competition, behavior, motive

## Abstract

**Background:**

Although runner’s profiles were previously investigated, information on the training frequency and training distance for short (5 km, 10 km) and long-distance (>21 km) running is absent. The present study aimed to investigate the associations between training routines and exercise habits of recreational endurance runners considering self-reported preferred race distance [10 km, half-marathon (HM), and marathon/ultra-marathon (M/UM)] subgroups.

**Methods:**

This is a cross-sectional study, sampling 154 recreational runners of both sexes. A web survey was used for data collection regarding age, sex, preferred distance (10-km, HM, M/UM), training routines, exercise habits, and periodized training routines. The Chi-square test (Cramer’s V) and Kruskal-Wallis test (Eta-Squared η^2^) with effect sizes were used for comparisons between race distances.

**Results:**

Significant differences were shown for anthropometric, training, and periodization characteristics. Highly significant differences were found between subgroups for the number of sessions, running kilometers, and training hours at all periods and within all four preparation conditions. M/UM runners were training more frequently, for longer durations, and ran greater distances each week.

**Conclusion:**

This finding supports the notion that training habits and periodization characteristics are different for different race distances (10-km, half marathon, marathon, and ultramarathon).

## Highlights

- The study highlights the existence of critical training differences between endurance runners based on their preferred racing distance.
- M/UM were primarily motivated by performance reasons, while 10 km/HM runners were motivated for recreational purposes.
- Training load reduction occurred in the competitive period for HF and 10 km runners, following tapering principles in training periodization.

## Background

The global trends in sports and physical activities emphasize running as one of the main activities worldwide (Hulteen et al., 2017). In long-distance running, the number of marathoners (Thuany et al., 2022a; Yang et al., 2022) and ultra-marathoners (Thuany et al., 2022a) has considerably increased in recent decades, with the motivations for training changing according to the distance (Waśkiewicz et al., 2019a,b). In marathon running, women focus more on body weight, life meaning, and self-esteem compared to men (Waśkiewicz et al., 2019b), whereas ultra-marathoners reported higher scores in affiliation and life meaning, and lower scores for weight concern, personal goal achievement, and self-esteem (Waśkiewicz et al., 2019a). Comparison between race distances showed that ultra-runners indicated a lower health and weight-oriented motivation than half-marathoners and marathoners (Gerasimuk et al., 2021).

Understanding runner motivation is an important topic given that it is related to the differences in time spent in training, including frequency, weekly training volume, the number of weekly training sessions, and participation in competitions and strategy for training periodization (Tanous et al., 2022). Similarly, the training commitment is related to improvement in health and psychological wellbeing (León-Guereño et al., 2020), with health outcomes ranging according to running distance. A recent meta-analysis showed a reduction of body mass, resting heart rate, and triglycerides with significantly increased cardiorespiratory fitness and high-density lipoprotein cholesterol after one year of running training (Junior et al., 2015). In contrast, a previous study highlights that for runners competing in the Santiago Marathon, those registered for the marathon have a higher risk of running injuries, compared to runners competing in 10 km (Besomi et al., 2019). Ultra-marathon running might have detrimental effects on health such as musculoskeletal problems, changes in cardiac biomarkers, impairment of liver and renal function, digestive disorders, and infections of the upper airways (Knechtle and Nikolaidis, 2018).

In this sense, race distance, runners’ profile, and running characteristics have different effects on training commitment as well as health. These differences, in association with different profiles, motivations, and training backgrounds (Besomi et al., 2018), highlight the need for tailored training strategies that consider the specific needs and the runner’s purpose. Despite this, information on training commitment and periodization for short and long-distance runners is lacking (Elbe et al., 2010; Whitehead et al., 2022). Addressing this challenge is an important point for researchers since it permits the development of effective training strategies considering runners’ needs as well reduces the rate of dropouts during a race and lowers the occurrence of injuries. Therefore, this is the first study to investigate the associations between training routines and exercise habits of recreational endurance runners considering 10 km, half-marathon (HM), and marathon/ultra-marathon (M/UM) race distance subgroups. Based on the results of prior studies on the training behaviors of runners over specific distances (Helgerud et al., 1990; Billat et al., 2003; Friedrich et al., 2014; Thuany et al., 2020; Knechtle et al., 2021), it was assumed that there would be critical training differences between endurance runners based on their preferred racing distance (Helgerud et al., 1990; Billat et al., 2003; Friedrich et al., 2014; Thuany et al., 2020; Knechtle et al., 2021).

## Materials and methods

### Study protocol and ethics approval

The Nutrition and Running High Mileage (NURMI) Study protocol (Wirnitzer et al., 2016) was accepted by the St. Gallen, Switzerland ethics board on May 6, 2015 (EKSG 14/145). The trial registration number is ISRCTN73074080 (retrospectively registered). Detailed information about NURMI Study Step 2 methodology has been described in previous publications (Boldt et al., 2019; Knechtle et al., 2021; Motevalli et al., 2022; Tanous et al., 2022; Wirnitzer et al., 2022).

### Participants

Based on three steps, the NURMI (Nutrition and Running High Mileage) Study was conducted with a cross-sectional study design (Wirnitzer et al., 2016). The primary participants were recruited from Austria, Germany, and Switzerland and were contacted mainly by social media platforms, websites of marathon event organizers, online running forums, email subscriptions and runner magazines, including magazines for health, nutrition, or lifestyle, sports trade fairs, plant-based diet and lifestyle, and personal contacts. The characteristics of the endurance runner participants are presented in Table 1.

**Table 1:** Characteristics of endurance runners displayed by race distance.

		Total	10 km	HM	M/UM	Statistics
		100% (245)	37% (91)	36% (89)	27% (65)	
Sex	Female	58% (141)	74% (67)	55% (49)	38% (25)	
	Male	42% (104)	26% (24)	45% (40)	62% (40)	
Age (years)		39 (IQR 17)	37 (IQR 18)	37 (IQR 18)	44 (IQR 17)	*F*_(2, 242)_ = 4.87 η^2^ = 0.04 *p* = 0.008
Body weight (kg)		65 (IQR 14.2)	62 (IQR 11)	65 (IQR 13)	67.5 (IQR 17.5)	*F*_(2, 242)_ = 5.05 η^2^ = 0.04 *p* = 0.007
Body height (m)		1.7 (IQR 0.1)	1.7 (IQR 0.1)	1.7 (IQR 0.1)	1.8 (IQR 0.1)	*F*_(2, 242)_ = 5.04 η^2^ = 0.04 *p* = 0.007
BMI (kg/m^2^)		21.7 (IQR 3.5)	21.3 (IQR 3.94)	22 (IQR 3.28)	22.2 (IQR 3.25)	*F*_(2, 242)_ = 1.22 η^2^ = 0.01 *p* = 0.296
Educational background	Unqualified	< 1% (1)	/	1% (1)	/	χ^2^_(8)_ = 11.46 V = 0.08 *p* = 0.177
	A-Levels or Similar	22% (53)	25% (23)	27% (15)	23% (15)	
	Upper Secondary Education/Technical Degree/GCSE or Similar	34% (83)	26% (24)	37% (33)	40% (26)	
	University or Higher Degree (i.e. doctorate)	34% (83)	42% (38)	30% (27)	28% (18)	
	N/A	10% (25)	7% (6)	15% (13)	9% (6)	
Nationality	Germany	72% (177)	74% (67)	73% (65)	69% (45)	χ^2^_(6)_ = 4.47 V = 0.06 *p* = 0.614
	Austria	18% (44)	18% (16)	17% (15)	20% (13)	
	Switzerland	5% (13)	2% (2)	7% (6)	8% (5)	
	Other Country	4% (11)	7% (6)	3% (3)	3% (2)	
Exercise motive	Health	9% (23)	14% (13)	8% (7)	5% (3)	χ^2^_(4)_ = 21.37 V = 0.17 *p* < 0.001
	Recreation	54% (133)	57% (52)	64% (57)	37% (24)	
	Performance	36% (89)	29% (26)	28% (25)	58% (38)	
Original motive to run	Health	44% (108)	46% (42)	45% (40)	40% (26)	χ^2^_(2)_ = 0.62 V = 0.05 *p* = 0.732
	Recreation	56% (137)	54% (49)	55% (49)	60% (39)	
Present motive to run	Health	19% (47)	22% (20)	20% (18)	9% (14)	χ^2^_(4)_ = 2.85 V = 0.06 *p* = 0.583
	Recreation	46% (113)	41% (37)	47% (42)	52% (34)	
	Performance	35% (85)	37% (34)	33% (29)	34% (22)	

Results are presented in percentages (%), total numbers, and median (IQR). χ^2^ statistic calculated by Pearson’s Chi-squared test and *F* statistic calculated by Kruskal-Wallis test. kg, kilogram; m, meters; A-Levels, Advanced level qualification; N/A, no answer; 10 km, 10 kilometers distance; HM, half-marathon distance; M/UM, marathon/ultra-marathon distance.

### Procedures

#### Experimental approach

The participants filled out an online survey for the NURMI Study Step 2 available from February 1 (2015) to December 31 (2015) in German and English languages at www.nurmi-study.com. Participants were given a written procedural description and provided their informed consent to participate in the study before filling out the questionnaire on physical/psychological health, which included the basic assignment to a sports area, the motivations of running, racing, and exercise activities, and participation in parallel sports to running in order to improve differentiation between a predominantly health, leisure or performance-orientated strategy to running within our sample of exclusively recreational runners.

For successful participation in the study, the following inclusion criteria must have been fulfilled: (1) completion of the informed consent (written), (2) 18 years of age or older, (3) survey of Step 2 completed, (4) successful participation in a running event of half-marathon or longer distance in the previous two years. Additionally, (5) participants were required to choose a (preferably long distance) race for the NURMI running event (a half-marathon (HM) or marathon (M) distance) to prepare and finish for Step 3 (the main NURMI Study, which linked Step 2 to Step 3) (Wirnitzer et al., 2016).

**Figure 1:**
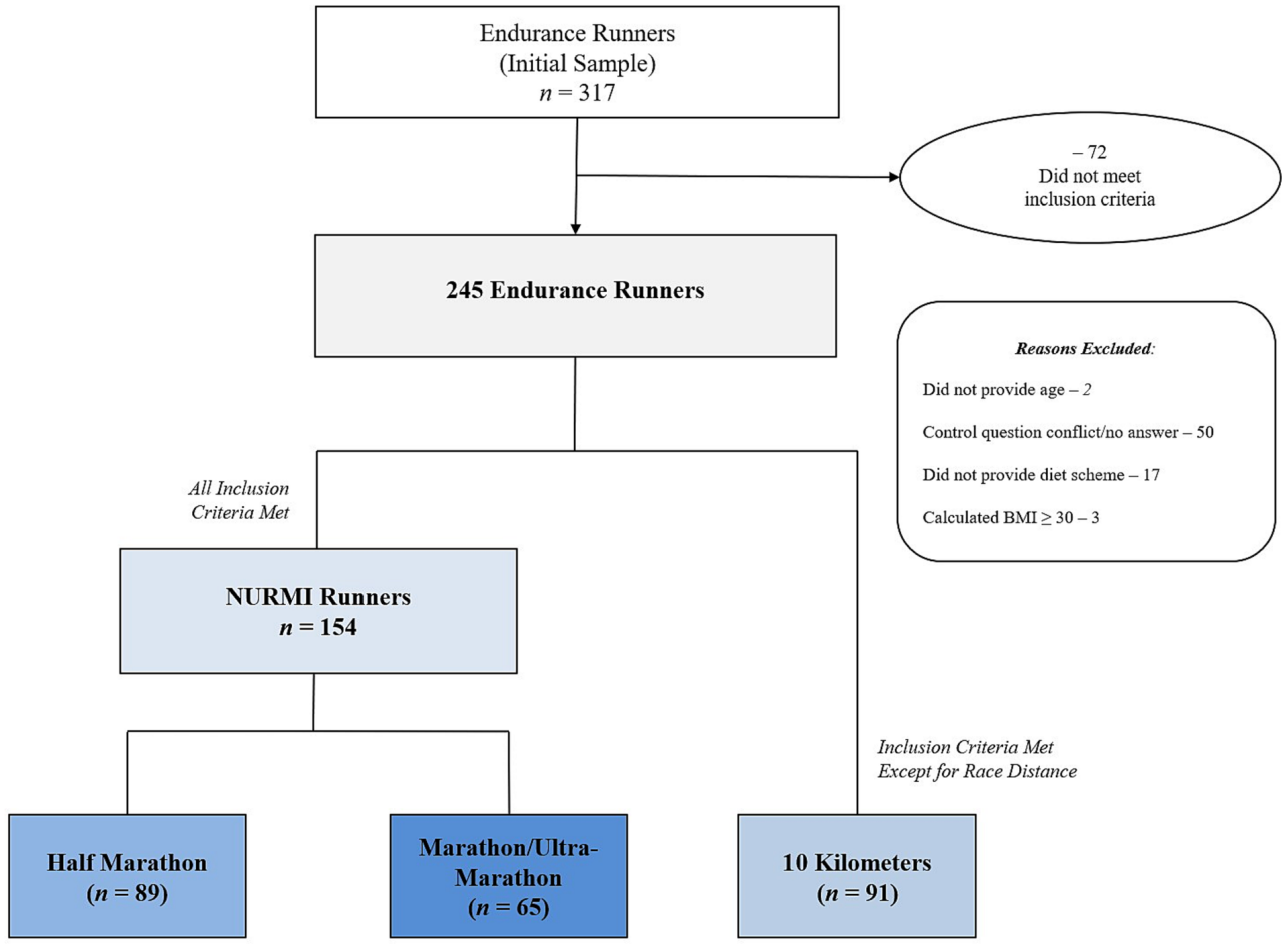
Enrollment and categorization of participants by race distance.

A total of 91 runners with high motivation gave accurate and useful answers for high-quality data. However, these 91 participants had not completed a half-marathon or marathon but had completed a 10-km race. To avoid the irreversible forfeit of their valuable data, 10-km runners who met all inclusion criteria but reported a 10-kilometers (10 KM) event as their main running event were included as an additional subgroup. Figure 1 shows participants’ categorization according to race distance subgroups, including 10 km, half-marathon, and marathon/ultra-marathon (data were grouped since the marathon distance is included in an ultra-marathon). The shortest ultra-marathon distance reported was 50 km, and the longest distance reported was 160 km. In addition, the interested reader is kindly directed to the sequential Part B paper and the associations of training and exercise routines with race performances (Knechtle et al., 2023).

#### Data clearance

To control for (i) running participation (history, experience, motives, training, or racing, etc.) and (ii) diet measures, two groups of control questions were included, each within different sections of the survey. In total, 72 participants were removed from the data analysis and excluded. Furthermore, in order to control for a minimal health status related to a minimum fitness level, and to further improve the reliability of data sets, the body mass index (BMI) approach following the World Health Organization was used (Lattice, 2008; World Health Organization (WHO), 2010a,b). However, with a BMI ≥ 30 kg/m^2^, and to safely reduce body weight first, other health protecting and/or weight loss approaches than running are necessary to minimize health risks. Three participants were excluded from data analysis due to having a BMI ≥ 30 kg/m^2^. In total, 317 endurance runners completed the questionnaire. Incomplete and inconsistent or conflicting data sets were removed and excluded from data analysis. After data clearance, 245 runners in total with complete data sets were included for the descriptive statistical analysis (shown in Figure 1).

#### Measures

Training routines and exercise habits of endurance runners active in running events were described by the following items regarding race distance: original and present motives to run (health, recreation, performance); favorite season and time of day to run (outdoors, indoors); total training timespan (duration preparing for the main race); training guidance (unsupervised, professional, or alternative); parallel sports participation (winter sports, summer sports); periodized training routines, including volume (weekly running sessions, average weekly and daily breadth of training (km, hours)) linked with training period and preparation conditions (Hulteen et al., 2017; Waśkiewicz et al., 2019b; Thuany et al., 2022a; Yang et al., 2022).

### Statistical analysis

All statistical analyses were performed with the statistical software R, version 3.6.2 Core Team 2019 (R Foundation for Statistical Computing, Vienna, Austria). Due to the exploratory nature of the study, the statistical tests were conducted using univariate methods. Data are presented as arithmetic mean and standard deviation for metric variables, median and interquartile range (IQR) for ordinal variables, and absolute/relative frequencies for categorical data. Significant differences (*p* ≤ 0.05) in running activity (training routines and exercise habits, etc.) between race distance subgroups were calculated by using a non-parametric test. Chi-square test (χ^2^; nominal scale; Cramer’s V strength of association: 0 – no association, 0.1–0.3 – weak association, 0.3–0.5 – moderate association, >5 – strong association) was used to examine the association between the variables, Kruskal-Wallis test (ordinal and metric scale; Eta squared (η^2^) measure of effect size: 0.01–0.05 – small effect, 0.06–0.13 – medium effect, ≥0.14 – large effect) approximated by using the *F* distributions. Differences in weekly and daily training with kilometers covered by race distance subgroups are displayed by various box plots using the lattice package in R (Lattice, 2008). The level of statistical significance was set at *p* ≤ 0.05.

## Results

In total, 317 distance runners completed the survey, of which 245 (141 women and 104 men) remained after data clearance with a median age of 39 (IQR 17) years, body weight of 65 kg (IQR 14.2), BMI of 21.7 kg/m^2^ (IQR 3.5) from Austria (*n* = 44), Germany (*n* = 177), Switzerland (*n* = 13) and some additional countries (*n* = 11: Belgium, Brazil, Canada, Italy, Luxemburg, Netherlands, Poland, Spain, United Kingdom). The final sample included 154 NURMI runners (89 competing at HM, 65 competing at M/UM) and 91 runners competing over the 10 km distance.

Race distance subgroups were found to differ significantly in age (*p* = 0.008), with M/UM runners being the oldest (44 years; IQR 17). For anthropometrics, significant differences were found based on race distance for body weight (*p* = 0.007), where 10 km runners were the lightest weight (62 kg; IQR 11), and height (*p* = 0.007), where M/UM runners were the tallest (1.8 m; IQR 0.1). No significant differences between race distance subgroups were observed in BMI (*p* = 0.296). Concerning the educational background of the participants, no significant difference was found across race distance subgroups (*p* = 0.177), most participants held an A-Levels (or similar degree) (22%; *n* = 53), an upper secondary school/technical education degree (34%; *n* = 83), or a university degree (34%; *n* = 83), while 1 (< 1%) was not qualified and 25 (10%) did not answer. A significant difference was found in the motive for exercising between the race distance subgroups (*p* < 0.001): 10 km runners were the most prevalent for health (14%; *n* = 13), HM runners for recreation (64%; *n* = 57), and M/UM runners for performance (58%; *n* = 38). Concerning the runners’ civil status, no significant difference was found for race distance (*p* = 0.744): 66 (27%) were single, 164 (67%) were married (or living with their spouse), and 15 (6%) participants were divorced or separated. Characteristics of the distance runners are provided in Table 1, and further specifics of these participants are given in the Part B arrangement (Knechtle et al., 2023).

No significant differences were identified by race distance subgroups for the original motive to run (*p* = 0.732) or the present motive to run (*p* = 0.583). The total sample most frequently reported recreation for their original motive (56%; *n* = 137) and present motive (46%; *n* = 113) to run. No significant differences were identified for favorite outdoor (*p* = 0.171) or indoor running season (*p* = 0.389), or for their favorite time of day, whether outdoor (*p* = 0.253) or indoor (*p* = 0.711) running. The total sample mostly preferred outdoor running in the spring (60%; *n* = 145) and in the morning (31%; *n* = 75), and indoor running in the winter (22%; *n* = 53) during the early evening (14%; *n* = 33).

Table 2 displays the participants’ macro exercise habits, such as the total training timespan, training guidance, and parallel sports participation by race distance subgroups. For the total training timespan (*p* = 0.637) and training guidance (*p* = 0.369), no significant differences were found between race distance subgroups, and most of the total sample trained for an extent of three to four months (52%; *n* = 122) unsupervised (76%; *n* = 179). Parallel to running, participants reported their concurrence in winter sports (snowboarding 7% or skiing; alpine 14%, Nordic 11%, backcountry 4%) and summer sports (biking 53%, trail/hill running 31%, swimming 31%, hiking 31%, triathlon 19%). A significant difference was identified in trail/hill running participation (*p* = 0.001), with a positive increase in participation prevalence across distance subgroups and most frequently among M/UM runners (46%; *n* = 29). No other significant differences were identified in parallel sports participation based on race distance subgroups.

**Table 2:** Macro exercise habits, including the training timespan, guidance, and parallel sports by race distance.

	Total	10 km	HM	M/UM	Statistics
	100% (245)	37% (91)	36% (89)	27% (65)	
Training timespan for main race					
1–2 months	20% (46)	19% (17)	24% (21)	14% (8)	χ^2^_(10)_ = 7.92 V = 0.06 *p* = 0.637
3–4 months	52% (122)	48% (42)	56% (49)	53% (31)	
4–6 months	21% (48)	23% (20)	16% (14)	24% (14)	
7–8 months	4% (9)	5% (4)	3% (3)	3% (2)	
9–10 months	2% (5)	2% (2)	1% (1)	3% (2)	
> 12 months	2% (4)	3% (3)	/	2% (1)	
Training guidance					
Unsupervised	76% (179)	78% (69)	70% (62)	83% (48)	χ^2^_(4)_ = 4.28 V = 0.08 *p* = 0.369
Professional	15% (36)	15% (13)	20% (18)	9% (5)	
Alternative	8% (19)	7% (6)	9% (8)	9% (5)	
Parallel sport participation					
Alpine skiing	14% (34)	12% (11)	15% (13)	16% (10)	χ^2^_(2)_ = 0.49 V = 0.04 *p* = 0.784
Nordic skiing	11% (26)	8% (7)	12% (11)	13% (8)	χ^2^_(2)_ = 1.38 V = 0.08 *p* = 0.501
Backcountry skiing	4% (9)	/	7% (6)	5% (3)	χ^2^_(2)_ = 6.00 V = 0.16 *p* = 0.05
Snowboarding	7% (16)	5% (5)	9% (8)	5% (3)	χ^2^_(2)_ = 1.35 V = 0.07 *p* = 0.509
Biking	53% (130)	49% (45)	55% (49)	57% (36)	χ^2^_(2)_ = 1.02 V = 0.06 *p* = 0.6
Trail/Hill running	31% (75)	19% (17)	33% (29)	46% (29)	χ^2^_(2)_ = 13.25 V = 0.23 *p* = 0.001
Swimming	31% (75)	33% (30)	35% (31)	22% (14)	χ^2^_(2)_ = 3.05 V = 0.11 *p* = 0.218
Hiking	31% (75)	29% (26)	33% (29)	32% (120)	χ^2^_(2)_ = 0.37 V = 0.04 *p* = 0.831
Triathlon	19% (49)	18% (16)	21% (19)	17% (11)	χ^2^_(2)_ = 0.54 V = 0.05 *p* = 0.765

Results are presented as percentages (%) and total numbers. χ^2^ statistic calculated by Pearson’s Chi-squared test.

**Figure 2:**
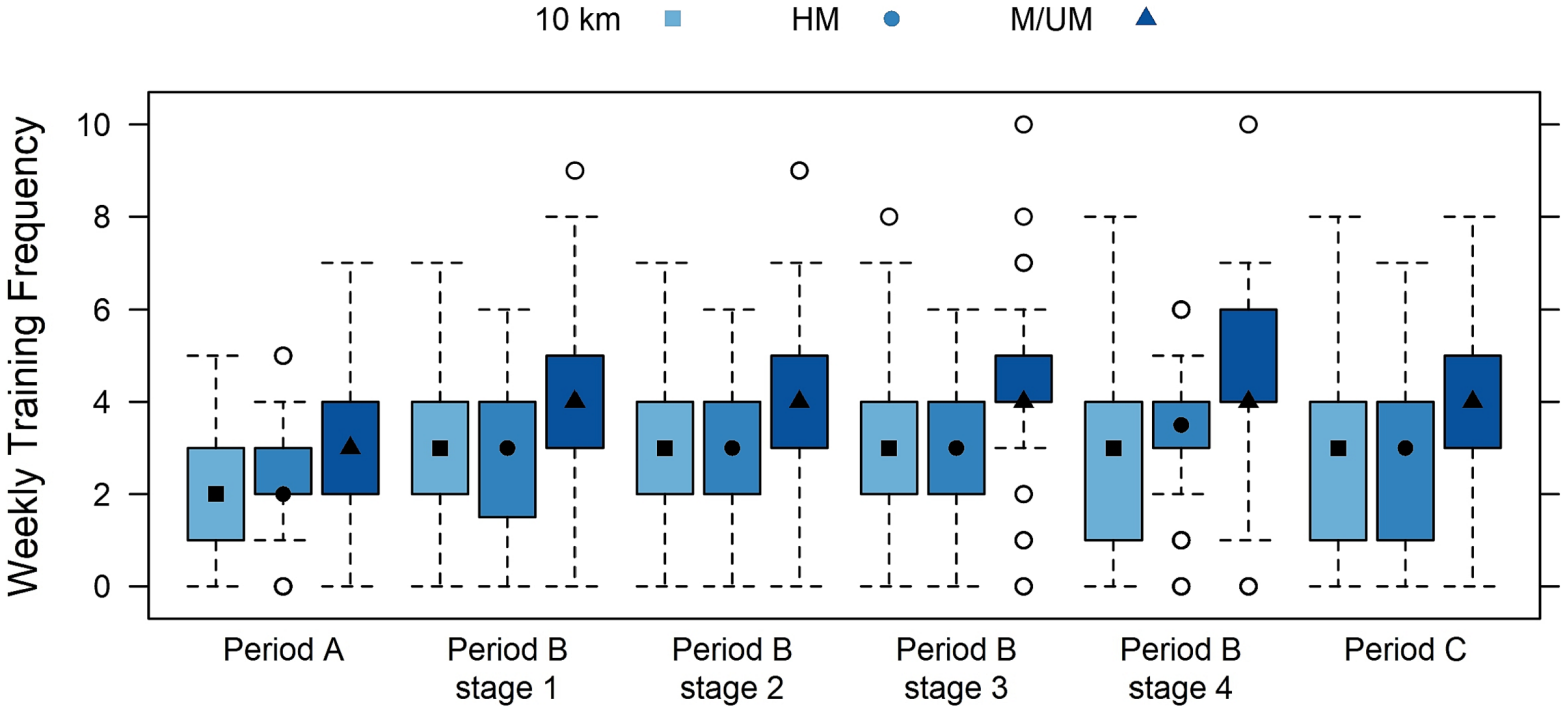
Box plots of interactions between race distance subgroups and weekly training sessions within training periods displayed by median (quartile range) including period B conditions.

**Figure 3:**
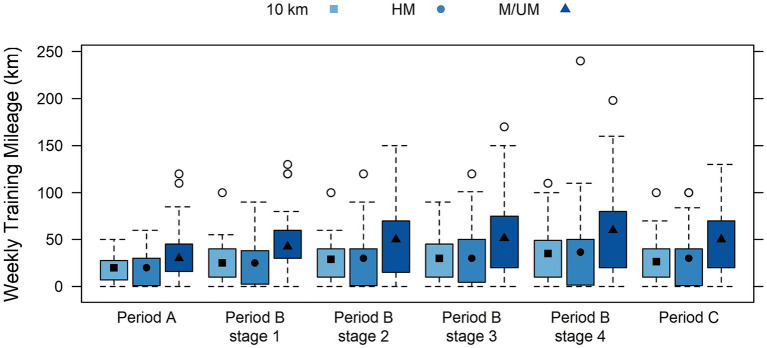
Box plots of interactions between race distance subgroups and weekly training kilometers within training periods displayed by median (quartile range) including period B conditions.

**Figure 4:**
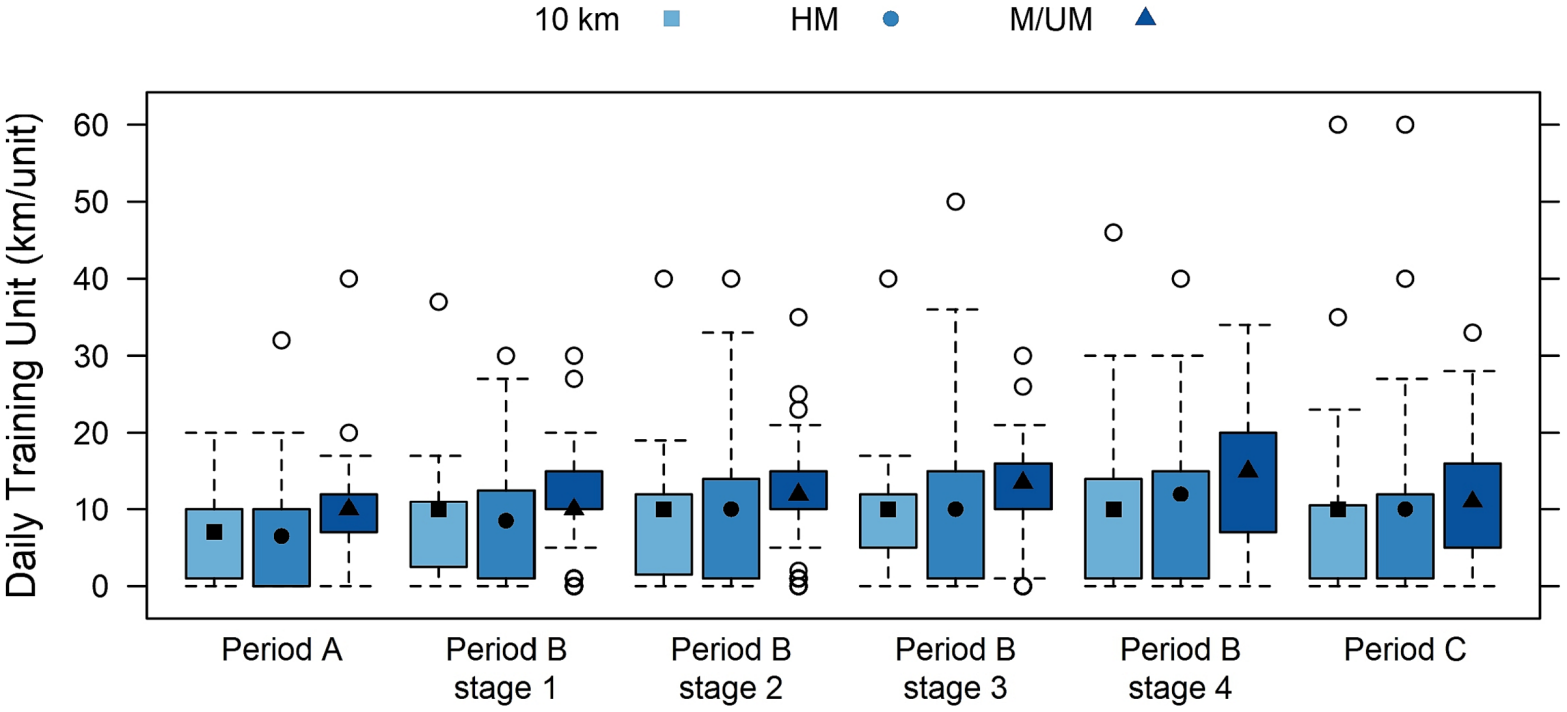
Box plots of interactions between race distance subgroups and daily training kilometers within training periods displaying median (quartile range) including period B conditions.

A training overview of the periodization phases for endurance runners is provided in Table 3 by race distance subgroups, including weekly sessions and weekly and daily distance covered in kilometers, and time spent training in hours. Highly significant differences were found between subgroups for all weekly variables (sessions, kilometers, hours) at all periods (A, B, C) and within all four preparation conditions (*p* < 0.001), where M/UM runners were found to train the most frequently for the longest distance and hourly durations each week. Race distance subgroup weekly training sessions within periodization phases are shown in Figure 2, and weekly kilometers are in Figure 3. Significant differences were found between the race distance subgroups in daily training kilometers covered and time spent training in Periods A and B, including every preparation condition within Period B (*p* < 0.01), and 10 km runners were always found to train over the least distance and time. Significant differences were found for race distance subgroups in daily training kilometers covered and time spent training in Period C (*p* < 0.05): HM runners ran the least distance (8.71 km ± 9.36), and 10 km runners spent the least amount of time training (0.38 h ± 0.37). Race distance subgroup daily training kilometers within periodization phases are shown in Figure 4.

**Table 3:** Periodization training routines, including frequency, mileages, and durations displayed by race distance.

		Total	10 km	HM	M/UM	Statistics
		100% (245)	37% (91)	36% (89)	27% (65)	
Period A – Transition & recovery phase						
Weekly	Sessions	3 (IQR 1)	2 (IQR 2)	2 (IQR 1)	3 (IQR 2)	*F*_(2, 219)_ = 9.52; η^2^ = 0.08; *p* < 0.001
	Kilometers	22.4 ± 18.9	18.5 ± 13.1	18.9 ± 15.6	32.7 ± 25.6		*F*_(2, 219)_ = 8.77; η^2^ = 0.07; *p* < 0.001
	Hours	1.21 ± 1.03	1.0 ± 0.71	1.02 ± 0.85	1.78 ± 1.39		*F*_(2, 219)_ = 8.70; η^2^ = 0.07; *p* < 0.001
Daily	Kilometers	7.06 ± 5.86	6.25 ± 4.42	6.4 ± 6.39	9.16 ± 6.49	*F*_(2, 219)_ = 6.02; η^2^ = 0.05; *p* = 0.003
	Hours	0.26 ± 0.21	0.23 ± 0.16	0.24 ± 0.23	0.33 ± 0.23		*F*_(2, 219)_ = 5.97; η^2^ = 0.05; *p* = 0.003
Period B – central preparation phase						
Preparation Condition 1 (fundamental training, general low intensity)						
Weekly	Sessions	3 (IQR 2)	3 (IQR 2)	3 (IQR 2)	4 (IQR 2)	*F*_(2, 219)_ = 11.98; η^2^ = 0.1; *p* < 0.001
	Kilometers	30.2 ± 24.6	25.3 ± 18.6	26 ± 22.8	43 ± 29.7		*F*_(2, 219)_ = 10.95; η^2^ = 0.09; *p* < 0.001
	Hours	4.63 ± 3.76	3.88 ± 2.85	3.99 ± 3.49	6.59 ± 4.56		*F*_(2, 219)_ = 11.26; η^2^ = 0.09; *p* < 0.001
Daily	Kilometers	8.74 ± 6.61	7.92 ± 5.85	8.1 ± 7.2	10.8 ± 6.46	*F*_(2, 219)_ = 5.28; η^2^ = 0.05; *p* = 0.006
	Hours	0.4 ± 0.3	0.36 ± 0.26	0.37 ± 0.33	0.49 ± 0.29	*F*_(2, 219)_ = 5.19; η^2^ = 0.05; *p* = 0.006
Preparation Condition 2 (expansive training with specialization, low or moderate intensity)						
Weekly	Sessions	3 (IQR 2)	3 (IQR 2)	3 (IQR 2)	4 (IQR 2)	*F*_(2, 219)_ = 10.26; η^2^ = 0.09; *p* < 0.001
	Kilometers	33.5 ± 27.5	27.4 ± 20.6	29.6 ± 25.7	47.7 ± 33.5		*F*_(2, 219)_ = 11.06; η^2^ = 0.09; *p* < 0.001
	Hours	4.82 ± 3.96	3.95 ± 2.96	4.26 ± 3.69	6.86 ± 4.82		*F*_(2, 219)_ = 11.48; η^2^ = 0.09; *p* < 0.001
Daily	Kilometers	9.42 ± 7.29	8.43 ± 6.23	8.8 ± 8.15	11.7 ± 7.08	*F*_(2, 219)_ = 6.86; η^2^ = 0.06; *p* = 0.001
	Hours	0.41 ± 0.32	0.37 ± 0.27	0.38 ± 0.35	0.51 ± 0.31		*F*_(2, 219)_ = 6.86; η^2^ = 0.06; *p* = 0.001
Preparation Condition 3 (interval training, pacing, specific competition focus, moderate or high intensity)						
Weekly	Sessions	4 (IQR 2)	3 (IQR 2)	3 (IQR 2)	4 (IQR 1)	*F*_(2, 219)_ = 7.92; η^2^ = 0.07; *p* < 0.001
	Kilometers	37.1 ± 31.1	30.2 ± 22.3	33.1 ± 28.5	52.7 ± 39.6		*F*_(2, 219)_ = 8.38; η^2^ = 0.07; *p* < 0.001
	Hours	5.65 ± 4.73	4.59 ± 3.39	5.04 ± 4.34	8.02 ± 6.01		*F*_(2, 219)_ = 8.71; η^2^ = 0.07; *p* < 0.001
Daily	Kilometers	9.98 ± 7.86	8.94 ± 6.28	9.64 ± 9.49	12 ± 7.17	*F*_(2, 219)_ = 5.90; η^2^ = 0.05; *p* = 0.003
	Hours	0.41 ± 0.32	0.37 ± 0.26	0.4 ± 0.39	0.49 ± 0.29		*F*_(2, 219)_ = 5.84; η^2^ = 0.05; *p* = 0.003
Preparation Condition 4 (competition trial, race-dependent focus, moderate or high intensity)						
Weekly	Sessions	4 (IQR 2)	3 (IQR 3)	4 (IQR 1)	4 (IQR 2)	*F*_(2, 219)_ = 11.20; η^2^ = 0.09; *p* < 0.001
	Kilometers	39.5 ± 35.8	31.8 ± 25	35.6 ± 35.7	56.2 ± 43.6		*F*_(2, 219)_ = 9.06; η^2^ = 0.08; *p* < 0.001
	Hours	5.95 ± 5.39	4.78 ± 3.76	5.36 ± 5.38	8.45 ± 6.57		*F*_(2, 219)_ = 9.15; η^2^ = 0.08; *p* < 0.001
Daily	Kilometers	10.7 ± 8.31	9.33 ± 7.62	10.2 ± 8.33	13.4 ± 8.74	*F*_(2, 219)_ = 6.39; η^2^ = 0.06; *p* = 0.002
	Hours	0.5 ± 0.39	0.44 ± 0.36	0.48 ± 0.39	0.63 ± 0.41		*F*_(2, 219)_ = 6.11; η^2^ = 0.05; *p* = 0.003
Period C – Main competitive phase (interim race stage/s & tapering)						
Weekly	Sessions	3 (IQR 2)	3 (IQR 3)	3 (IQR 3)	4 (IQR 2)	*F*_(2, 219)_ = 9.32; η^2^ = 0.08; *p* < 0.001
	Kilometers	32.2 ± 27.7	26 ± 20.5	27.8 ± 24.7	47.3 ± 34.8		*F*_(2, 219)_ = 9.17; η^2^ = 0.08; *p* < 0.001
	Hours	4.41 ± 3.8	3.57 ± 2.8	3.8 ± 3.37	6.48 ± 4.77		*F*_(2, 219)_ = 9.03; η^2^ = 0.08; *p* < 0.001
Daily	Kilometers	9.35 ± 8.7	8.79 ± 8.62	8.71 ± 9.36	11 ± 7.75	*F*_(2, 219)_ = 3.59; η^2^ = 0.03; *p* = 0.029
	Hours	0.41 ± 0.37	0.38 ± 0.37	0.38 ± 0.4	0.48 ± 0.33		*F*_(2, 219)_ = 3.37; η^2^ = 0.03; *p* = 0.036

Results are presented as median (IQR) and mean (SD). *F* statistic calculated by Kruskal-Wallis test. km – kilometer.

## Discussion

This study aimed to investigate exploratory associations in training routines and exercise habits between recreational endurance runners of self-reported race distance subgroups (10 km, HM, M/UM) with the assumption that that there would be critical training differences between endurance runners based on their preferred racing distance. The main findings were (i) M/UM runners had a greater body weight and body height than 10 km and HM runners but BMI was similar across race distance subgroups; (ii) M/UM runners were mostly exercise motivated for performance reasons, whereas 10 km and HM runners were mostly motivated to exercise for recreational purposes; (iii) no significant differences were found in the total training timespan or reports of training guidance based on race distance subgroups; (iv) weekly training sessions, kilometers, and hours were higher for M/UM runners across training periods and all preparation conditions of Period B; (v) HM runners ran the lowest daily distance in Period C, while 10 km runners spent the least amount of time training in Period C. While there are some comparable exercise habits within the training routines of endurance runners regardless of preferred distance, the overall results of the present investigation uphold the assumption that there are critical training differences between endurance runners based on their preferred racing distance.

### Differences in body dimensions

The first important finding was that M/UM runners had a higher body weight and height than 10 km and HM runners, but BMI was similar across race distance subgroups. In general, runners presented an average BMI (21.3 to 22.2 kg/m^2^) with similar values to those reported in previous studies with marathoners (19 to 21.8 kg/m^2^) (Tanda and Knechtle, 2013; Nikolaidis and Knechtle, 2020). Morphological characteristics were previously investigated in running studies (Knechtle et al., 2008; Sedeaud et al., 2014), with two main outcomes highlighted: performance and injuries. Previous studies show that higher values of BMI were related to a reduction in running speed (Knechtle and Nikolaidis, 2018), given the metabolic cost of body transport during running. For ultra-marathoners, BMI explained about 10 to 12% of the variance in running speed (Hoffman, 2008). In addition, BMI was related to training behaviors for performance prediction in different race distances (e.g., 5 km, 10 km, half-marathon, marathon) (Christou et al., 2021; Thuany et al., 2022b). BMI was also related to running-related injuries (Buist and Bredeweg, 2011), and needs to be carefully considered in association with training behaviors and motivation.

### Differences in motivation

A second important finding was a difference in the motivation to exercise. M/UM runners were mostly exercise motivated for performance reasons, whereas 10 km and HM runners were mostly motivated to exercise for recreational purposes, according with previous findings (Doppelmayr and Molkenthin, 2004; Hanson et al., 2015; Waśkiewicz et al., 2019a). Considering different performance levels (e.g., novice, recreational) and sub-groups (e.g., road-long distance, marathoners, ultra-marathoners, trail and track runners), runners competing in long-distance events presented higher scores for psychological goals and meaning of life and self-esteem compared to the health-orientation found in novice and recreational runners (Besomi et al., 2017). Ultra-marathoners focus more on the motives of ‘nature’ and ‘life meaning’ in their races compared to marathoners, focusing more on the importance of competition (Doppelmayr and Molkenthin, 2004).

Previous studies also showed that novice runners tend to be engaged in short distances (Malchrowicz-Mosko et al., 2020; Manzano-Sánchez et al., 2020) and start running for health motives, which can be related to the present findings since weekly training sessions, kilometers, and hours were higher for M/UM runners across training periods. In addition, despite the fact that the present study was not primarily focused on sex differences, a potential factor to explain the results includes the unbalanced distribution of men and women among the race distances. A higher frequency of male runners among the M/UM group (62%) can be related to the higher frequency of runners motivated for performance reasons. These differences were investigated previously, with men being more competition motivated and women presenting higher scores for coping, self-esteem, and goal achievement (Nikolaidis et al., 2019; Manzano-Sánchez et al., 2020).

### Differences in training

A further important finding was that M/UM runners showed higher indicators of training characteristics (i.e., weekly training sessions, running kilometers, and training hours) across training periods and all preparation conditions of Period B (central preparation phase). Similarly, a previous finding showed that marathoners presented a higher weekly training distance, training frequency, and longest endurance run before the running event (Fokkema et al., 2020) compared with half-marathoners. However, to the best of our knowledge, few studies were developed to understand the training periodization characteristics in recreational runners (Casado et al., 2022; Tanous et al., 2022). Most of the previous studies were developed to understand the patterns of endurance in elite athletes (Casado et al., 2022), which means that comparisons are difficult.

Furthermore, no differences were found in the total training timespan or reports of training guidance based on race distance subgroups. These results agree with previous findings, where running was considered one of the main physical activities performed across the world (Hulteen et al., 2017), which means that regardless of the distance, runners tend to be engaged in running for a long time without professional supervision. Prevalence statistics about professional supervision in runners need to be considered in future studies.

The training load reduction in Period C for both HM and 10 km runners is according to the tapering in training periodization, since period C concentrates on the competitive period (Haugen et al., 2022). That is, a markedly reduced weekly training load for subgroups was expected compared to earlier phases. However, the lack of information about the percentual reduction of training volume during the weeks, the training intensity, and training methods impair the generalization of the present findings.

### Limitations, practical applications, and implications for future research

The primary limitation of this exploratory investigation to be considered is the cross-sectional design, which is like other studies that are based on self-report (Besomi et al., 2017; Deelen et al., 2019). Thus, over- and under-reporting of the results are plausible based on the participants’ social expectations or a subjective understanding of sport science discipline-specific terminology. Previous studies also showed good reproducibility of runners’ self-reporting anthropometric variables and training characteristics (Nikolaidis and Knechtle, 2020). However, control questions were implemented throughout the questionnaire to minimize reporting errors. Therefore, the results obtained include a few limitations to be addressed for a careful interpretation of the findings. The sample size was small, considering that running is a relatively common sport. Furthermore, there was an unequal distribution of participants within the race distances *per se* (i.e., 27% of the total sample being M/UM runners vs. 37% being 10 km runners). However, most participants in this study were competing in the HM distance or marathon/ultra-distances. Finally, the apprehension of some findings may apply only to Western/European runners and these cultures, as most participants were from Austria, Switzerland, and Germany. This study can be used to better understand human behavior in the context of running training as well as to ascertain the best strategy to maintain involvement. Future studies should consider the impact of different race distances on physical, motor, and mental health improvements. To better understand runners’ motivations in different countries, natural (average temperature, wind, rain, snow) and built environments (parks, mountains, city characteristics) and their impact on training should be considered.

## Conclusion

In summary, differences exist for anthropometric, training, and periodization characteristics for different race distances (i.e., 10-km, half-marathon, marathon, and ultramarathon). The most important differences were found for the number of sessions, running kilometers, and training hours at all periods and within all four preparation conditions. Especially, marathon and ultra-marathon runners were training more frequently, for longer durations, and ran greater distances each week. This finding supports the notion that training habits and periodization characteristics are different for different race distances (10-km, half marathon, marathon, and ultramarathon).

## Data availability statement

The data sets generated during and/or analyzed during the current study and presented in this article are not publicly available. Requests to access the datasets should be directed to info@nurmi-study.com. Subjects will receive a brief summary of the results of the NURMI Study if desired.

## Ethics statement

The study protocol is available online via https://springerplus.springeropen.com/articles/10.1186/ s40064-016-2126-4 and was approved by the ethics board of St. Gallen, Switzerland on May 6, 2015 (EKSG 14/145). The study was conducted in accordance with the ethical standards of the institutional review board, medical professional codex, and with the 1964 Helsinki declaration and its later amendments as of 1996, the Data Security Laws, and good clinical practice guidelines. Study participation was voluntary and could be canceled at any time without the provision of reasons or negative consequences. Informed consent was obtained from all individual participants included in the study considering the data collected, used, and analyzed exclusively and only in the context of the NURMI Study for scientific publication.

## Author contributions

BK: Conceptualization, Study Design, Methodology, Writing – original draft, Writing – review & editing. DT: Formal analysis, Writing – original draft, Writing – review & editing. MT: Writing – original draft, Writing – review & editing. MM: Formal analysis, Writing – review & editing. GW: Data curation, Resources, Software, Writing – review & editing. CL: Conceptualization, Study Design, Methodology, Writing – review & editing. KWe: Writing – original draft, Writing – review & editing. TR: Writing – review & editing. KWi: Conceptualization, Data curation, Formal analysis, Methodology, Study Design, Writing – original draft, Writing – review & editing.
